# Treatment of Fingernail Onychomycosis with Efinaconazole 10% Solution in a Patient with Scleroderma: A Case Report

**DOI:** 10.1159/000522407

**Published:** 2022-03-09

**Authors:** Rhiannon C. Miller, Shari R. Lipner

**Affiliations:** Department of Dermatology, Weill Cornell Medicine, New York, New York, USA

**Keywords:** Fingernail fungus, Onychomycosis, Topical antifungal, Efinaconazole, Autoimmune disease

## Abstract

**Introduction:**

Oral antifungals are typically preferred over topicals for moderate to severe onychomycosis due to efficacy and shorter treatment courses. However, systemics are contraindicated or cautioned in patients with liver dysfunction and with some autoimmune diseases, and in those taking interacting medications. Efinaconazole 10% solution is a topical antifungal therapy, but application for fingernail onychomycosis has not been adequately studied.

**Case Presentation:**

We present a case of a 78-year-old female with scleroderma and moderate onychomycosis of the right 4th fingernail successfully treated with topical efinaconazole 10% solution.

**Conclusion:**

We review the literature on contraindications to oral antifungals for onychomycosis, precautions with terbinafine in patients with some autoimmune diseases, and topical onychomycosis therapies. Topical efinaconazole may represent an effective alternative for patients with fingernail onychomycosis who have contraindications to oral medications.

## Established Facts

- Topical efinaconazole 10% solution is an effective treatment for toenail onychomycosis but has not been well studied for fingernail onychomycosis.
- Alternative therapies are needed for patients with onychomycosis who have contraindications or precautions to taking oral antifungals.

## Novel Insights

- We demonstrate effective treatment of fingernail onychomycosis with efinaconazole 10% solution in a 78-year-old patient with scleroderma.
- Treatment with efinaconazole 10% solution should be considered for treatment of fingernail onychomycosis in older patients and those with contraindications to oral antifungals.

## Introduction/Literature Review

Efinaconazole 10% solution is a topical triazole antifungal that is approved by the United States Food and Drug Administration (FDA) for treatment of toenail onychomycosis due to *Trichophyton rubrum* and *T. mentagrophytes*. Treatment of fingernail onychomycosis with efinaconazole 10% solution has not been well studied.

For moderate to severe onychomycosis, treatment with oral antifungals is preferred due to generally better efficacy and shorter courses compared to topical therapy. However, treatment with oral antifungals may not be advised in onychomycosis patients with hepatic dysfunction, with some autoimmune diseases, or those taking interacting medications [1].

## Case Report

A 78-year-old female presented with yellow discoloration and lifting of the right 4th fingernail for the previous 3 months. Medical history was significant for scleroderma, hypertension, osteoarthritis, osteoporosis, iron-deficiency anemia, and gastroesophageal reflux. Medications included mycophenolate mofetil, atorvastatin, duloxetine, famotidine, olmesartan, omeprazole, raloxifene, iron, and calcium.

**Fig. 1:**
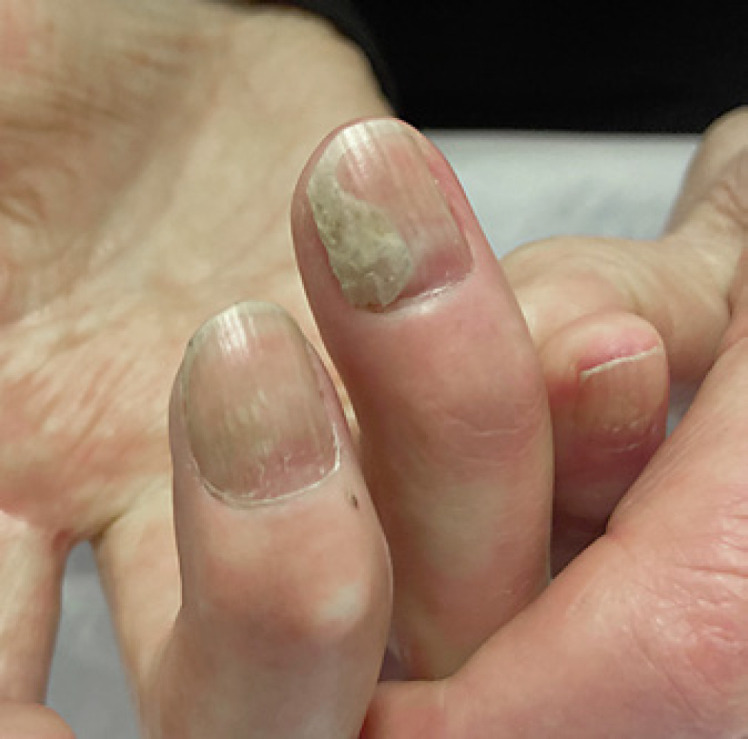
Right 4th fingernail on initial examination.

Clinical examination of the fingernails was significant for sclerodactyly, digital ulceration, and severe onycholysis of the right 4th fingernail (Fig. 1). Toenails were mildly yellowed and thickened.

Histopathology of fingernail clippings showed extensive infiltrating large septated hyphal forms, highlighted by PAS and GMS stains, and rare spores. Toenail clippings were negative for fungal and yeast elements. X-ray of the right hand was significant for diffuse osseous demineralization, degenerative changes at the wrist and triscaphe joints, and flexion deformity at the 2nd–5th proximal interphalangeal joints, related to known history of osteoporosis, osteoarthritis, and scleroderma, respectively.

**Fig. 2:**
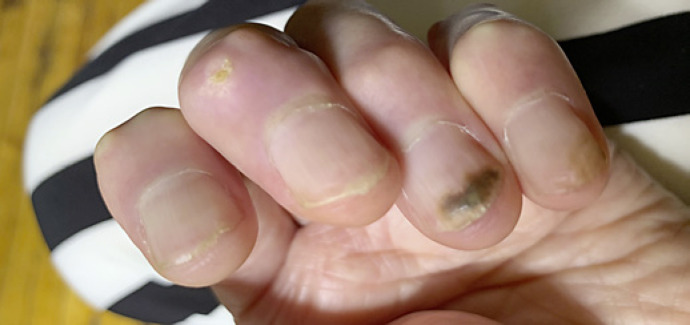
Clinical image after 3 months of topical efinaconazole use. Noted improvement in onycholysis, but new dark green discoloration of affected nail, consistent with concomitant *P. aeruginosa* infection.

**Fig. 3:**
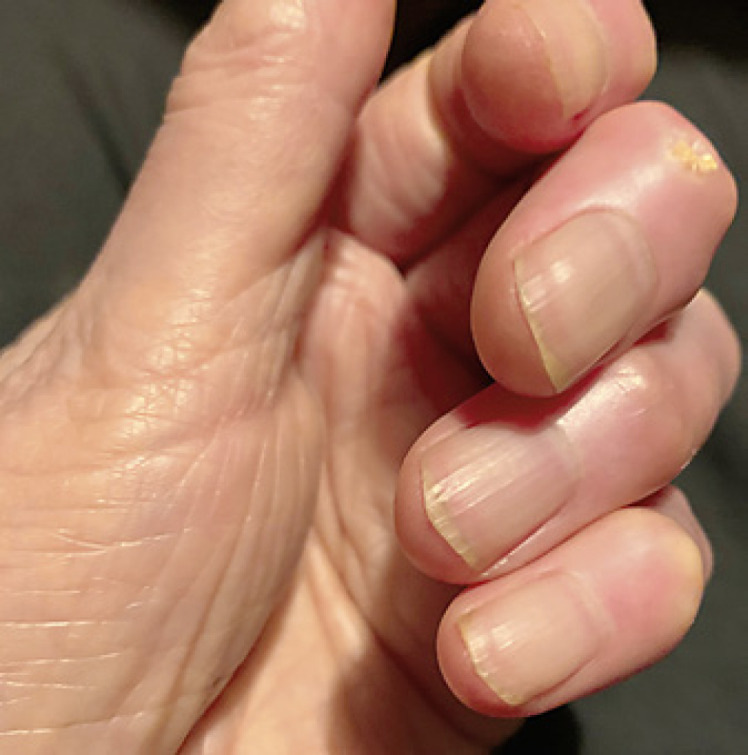
Clinical image after 6 months of topical efinaconazole use. Complete clinical improvement in nail appearance.

Onychomycosis severity index was 12, consistent with moderate onychomycosis. After a discussion of treatment options, the patient had reservations about taking systemics due to her autoimmune history and concomitant medications, and a decision was made to treat topically. She was prescribed topical efinaconazole 10% solution nightly. After 3-months of treatment, she presented with increased clear proximal nail growth. There was also green nail plate discoloration, consistent with concomitant *Pseudomonas aeruginosa* colonization (Fig. 2). The efinaconazole 10% solution was continued and gentamicin 0.3% solution was added for *P. aeruginosa* treatment. After 6 months of treatment with topical efinaconazole 10% solution, the affected nail exhibited clinical cure (Fig. 3).

## Discussion

Oral antifungals are effective treatments for moderate to severe onychomycosis but may not be appropriate for all patients [2]. For patients with hepatic dysfunction, terbinafine is contraindicated due to rare cases of hepatotoxicity. Since terbinafine is primarily eliminated by the kidney, caution is also advised for patients with renal disease. There are also some drug interactions with terbinafine through metabolization by CYP2D6, namely some tricyclic antidepressants (amitriptyline, clomipramine), SSRIs (paroxetine), antipsychotics (haloperidol, aripiprazole), antihistamines (cimetidine), immunosuppressants (cyclosporine), and antihypertensives (beta-blockers) [3]. Side effects with terbinafine are uncommon and drug-drug interactions typically cause only mild changes in serum drug levels but may cause anxiety amongst physicians and patients. Some serious medication interactions have been reported between oral itraconazole/fluconazole and common medications, such as this patient's atorvastatin. However, these interactions have only been reported with daily azole use, rather than the once weekly dosing regimen of fluconazole used for onychomycosis therapy [4].

While most patients with autoimmune diseases usually tolerate oral antifungals with no complications, some patients and their referring physicians may be anxious about taking antifungal systemics, due to side effects listed on the package insert. Terbinafine has been rarely associated with precipitation or exacerbation of cutaneous and systemic lupus erythematosus during post-marketing experience, and the package insert strongly cautions about use in patients with a known history of lupus [5]. Autoimmune disease is also a significant risk factor for terbinafine-associated erythema multiforme (EM), with 35% (6/17) of patients developing terbinafine-associated EM having a history of autoimmune disease, in a retrospective review [6]. Additionally, some autoimmune diseases are associated with hepatic disease, including nodular regenerative hyperplasia, hepatic vein thrombosis, drug-induced livery injury, fatty infiltration, and simultaneous autoimmune hepatitis [7]. Specifically, in scleroderma, 10% of patients have liver disease, most commonly primary biliary cholangitis [8].

For patients who cannot take or prefer not to take oral onychomycosis therapies, effective and safe topical treatments are needed. FDA-approved topical therapies include efinaconazole 10% solution, tavaborole 5% solution, and ciclopirox 8% lacquer. While clinical trials were not head-to-head and had different study designs and endpoints, efinaconazole appears to have the greatest efficacy with mycologic cure rates of 53.4–55.2% [9], compared to 31.1–35.9% for tavaborole and 29–36% for ciclopirox in their respective clinical trials [10, 11]. However, since none of the clinical trials enrolled patients with fingernail onychomycosis, efinaconazole is only FDA-approved for toenail onychomycosis. There has only been one study investigating efinaconazole treatment for fingernail onychomycosis (*N* = 10), with 50% of patients exhibiting complete clinical cure, 20% with marked improvement, 10% with improvement, and 20% with slight improvement (average treatment duration of 8.9 months) [12].

In this case, the patient had clinical cure with 6 months of efinaconazole treatment and no side effects. Topical efinaconazole is prescribed for 12 months for toenail onychomycosis. However, based on average fingernail growth rate of 2–3 mm/month, fingernail cure would be expected at 6 months, as was seen in this patient.

Green nail syndrome has not been reported in case reports, clinical trials, or product labeling for efinaconazole 10% solution. The concomitant *P. aeruginosa* colonization during efinaconazole treatment was likely unrelated to the drug. More likely the patient's onycholysis and immunosuppression, coupled with hand washing, predisposed her to *P. aeruginosa* colonization [13, 14]. The bacterial colonization resolved with gentamicin 0.3% solution while efinaconazole treatment was continued.

Additionally, in the phase III clinical trials of efinaconazole 10% solution for onychomycosis treatment, patients ages 75 and older were excluded. Our patient was 78-years-old with a favorable clinical outcome and no adverse events, demonstrating that efinaconazole treatment may be considered in older adults with onychomycosis. Older patients are more likely to have comorbidities or take medications that prevent them from taking oral therapy, so effective topicals are particularly necessary for this population.

## Conclusion

Topical efinaconazole 10% solution should be considered in the treatment of moderate fingernail onychomycosis in patients who have contraindications or precautions to taking oral antifungals.

## Statement of Ethics

Since this study was a case report, ethics approval was not required by the Weill Cornell Medicine Institutional Review Board. Written informed consent was obtained from the patient for publication of this case report and any accompanying images.

## Conflict of Interest Statement

Rhiannon Miller has no conflicts of interest. Dr. Lipner has served as a consultant for Ortho-dermatologics, Verrica, Hexima, and Hoth Therapeutics.

## Funding Sources

This article has no funding source.

## Author Contributions

Rhiannon Miller contributed to chart review, literature review, consenting the patient, and writing/revision of the manuscript. Dr. Lipner contributed to the diagnosis/treatment of the patient, chart review, literature review, and writing/revision of the manuscript.

## Data Availability Statement

All data generated or analyzed during this study are included in this article. Further enquiries can be directed to the corresponding author.

## Notes

This work and images have not been previously submitted or published.
